# The gut-brain axis and inflammatory mediators in suicide and mental disorders with high suicide rates: a review of current evidence

**DOI:** 10.1080/19585969.2026.2636468

**Published:** 2026-04-10

**Authors:** Ahmad Shamabadi, Razman Arabzadeh Bahri, Melika Arab Bafrani, Hanie Karimi, Hassan Asadigandomani, Hamed Vahidi, Shahin Akhondzadeh

**Affiliations:** ^a^ Psychiatric Research Center, Roozbeh Psychiatric Hospital, Tehran University of Medical Sciences, Tehran, Iran; ^b^ School of Medicine, Tehran University of Medical Sciences, Tehran, Iran

**Keywords:** Brain-gut axis, digestive system, enteric bacteria, intentional self injury, intestinal microflora, suicidal ideation

## Abstract

The interplay between gastrointestinal microbiota and mental disorders has recently been spotlighted. This review investigated discrete evidence suggesting associations between the gastrointestinal microbiome and inflammation with suicide. Fusicatenibavter, Hungatella, Veillonella, and Megasphaera have positive associations, but Clostridium, Butyricicoccus, Desulfovibrio piger, and Parabacteroides merdae have negative associations with suicidality. Additionally, lower species uniformity index, higher intestinal fatty acid binding protein secretion, lower zonulin secretion, higher interleukin-6 in cerebrospinal fluid, and laxative abuse are associated with suicidality. As nearly 90% of suicides occur in patients with mental disorders, the interaction between the gut microbiota and inflammation with these disorders together was also documented. Regarding this, major depressive disorder, psychosis and schizophrenia, generalised anxiety disorder, and substance use disorder were investigated. Bacteroidetes and Firmicutes show prominent changes in most cases. In addition, gut bacterial and non-bacterial microbiome alterations and subsequent dysbiosis may contribute to inflammation, in which cytokines affect microglial activity. Meanwhile, impaired intestinal homeostasis may influence these disorders through the vagus nerve, the hypothalamus-pituitary-adrenal axis, and the kynurenine pathway. Beyond these, direct effects of the gut microbiome on immunity are being hypothesised. In conclusion, the gut microbiota imbalance may influence the nervous system environment from non-inflammatory to inflammatory caused by pro-inflammatory cytokine influx into the brain. Consequently, microbiota imbalances may be associated with mental disorders. Specifically, limited evidence indicated possible links between microbiome alterations and suicide, highlighting the need for further research clarifying these associations and underpinning mechanisms. Other factors, including genetic vulnerability, environmental influences, and neurochemical pathways, should also be considered.

## Introduction

Intentional self-harm resulting in premature death is a major public health problem. More than 800,000 people worldwide and 47,646 people in the United States died by suicide in 2021 (National Center for Health 2021; Sher and Oquendo 2022), and there are 10 to 40 suicide attempts for every death by suicide (Chang et al. 2011; Sher and Oquendo 2022). In addition, each suicide is a unique tragedy that includes many people in its circle of negative influence (Cerel et al. 2019). Suicide is the second leading cause of death among people in the second and third decades of life in both genders worldwide (National Center for Health 2021). Its annual economic burden on the United States society is estimated at $70 billion, considering lifetime medical and work-loss costs (Stone et al. 2021).

The interplay between the gastrointestinal microbiome and physical and mental disorders has recently been in the spotlight (Järbrink-Sehgal and Andreasson 2020). A strong connection has been revealed between gut health and mental well-being, emphasising the role of microbial dysbiosis and dietary patterns in psychological outcomes (Khan et al. 2024). Emerging evidence has suggested that the gastrointestinal microbiome interacts with the central nervous system (CNS) through effects on nerves, hormones, and the inflammatory system (Eisenstein 2016). An increasing number of studies have shown that the gut microbiome has a special role in mental disorders; the gut-brain axis affects cognition and emotions, and the microbiome influences behaviour and mood (Eisenstein 2016; Järbrink-Sehgal and Andreasson 2020). Findings suggested that individuals with gastrointestinal disorders are more likely to experience higher levels of anxiety and depression (Khan et al. 2024). In addition, it has been shown that patients with bipolar disorder have disrupted brain activity in specific regions, accompanied by increased inflammatory markers and gut microbial imbalances. Research in this regard also suggested that Toxoplasma gondii infection may influence decision-making and risk-taking behaviour, potentially increasing suicide risk; however, limited evidence indicated possible links between microbiome alterations and suicidal behaviour (Zerekidze et al. 2024). Notably, interactions between gut bacteria and inflammatory cytokines were possibly linked to brain function changes (Guo et al. 2024). The benefits of related treatments have reinforced these findings. Certain dietary components, such as probiotics, prebiotics, synbiotics, postbiotics, dairy products, spices, fruits, vegetables, and medicinal herbs, may help protect against mental disorders by promoting beneficial gut microbiota and inhibiting harmful ones (Xiong et al. 2023).

While the gut microbiome and inflammation have been implicated in mental disorders, evidence directly linking them to suicide and self-harm remains limited and inconclusive. This review aims to document and review (i) associations between the gastrointestinal microbiome and inflammation with self-harm and suicide and (ii) the interaction among the gastrointestinal microbiome, the inflammatory system, and the most prominent mental disorders leading to suicide together for the first time considering a scale for the quality assessment of narrative review articles (SANRA) (Baethge et al. 2019). To achieve the first aim, the string “(gut OR microbio*) AND (suicid* OR “self harm” OR “self injury”)” was searched in PubMed, and to achieve the second, related data were extracted from the reviews using the terms gut and microbio* and the keywords associated with that mental disorder, and then, the references of the included reviews. Original clinical and preclinical studies, particularly with an emphasis on inflammation, were of interest.

## Microbiome, inflammation, and self-injury/suicide

**Figure 1.**
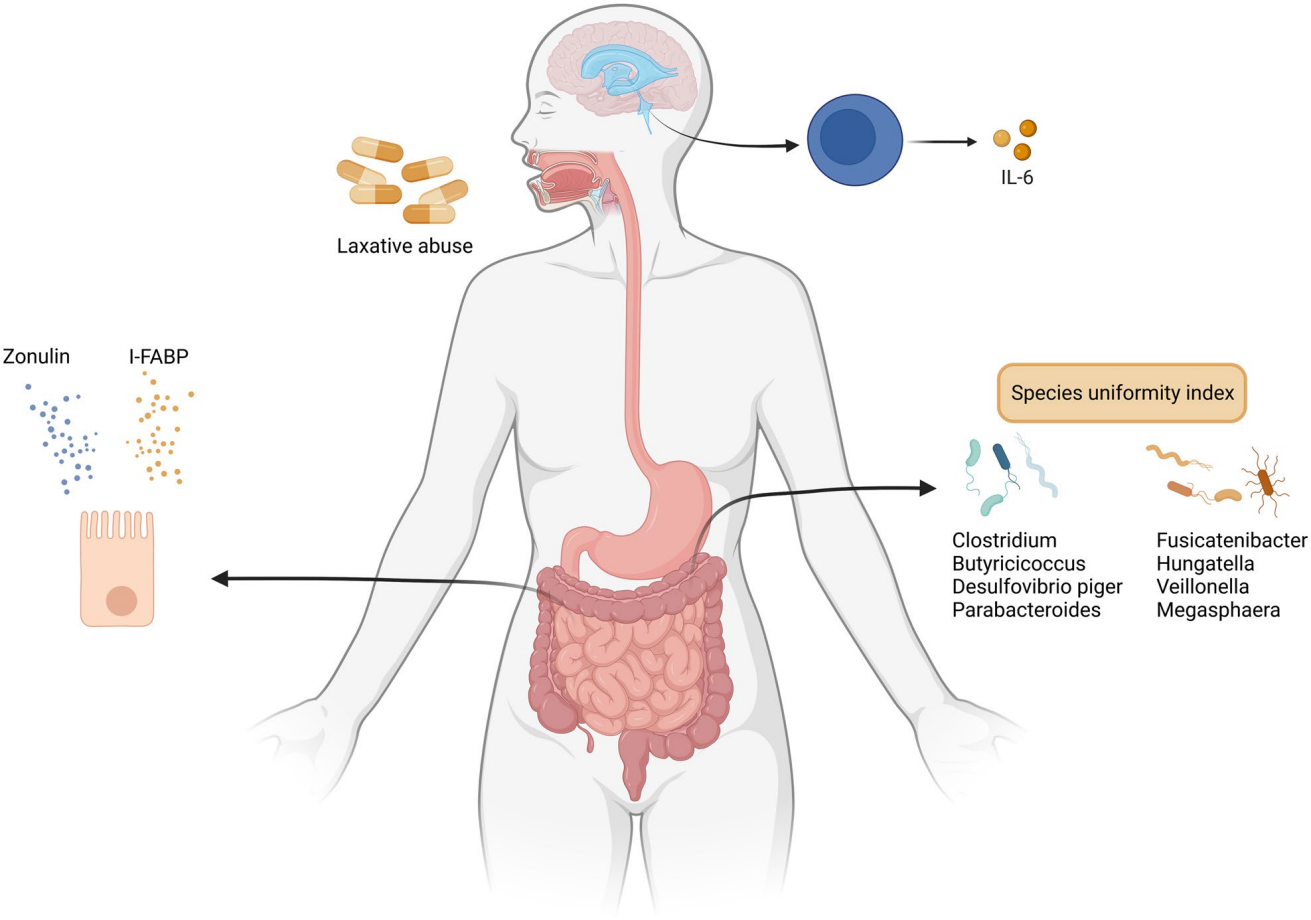
Some bacteria have positive associations, while others have negative associations with suicidality. In addition, lower species uniformity index, higher intestinal fatty acid binding protein secretion (I-FABP), lower zonulin secretion, higher interleukin (IL)-6 in cerebrospinal fluid, and laxative abuse are associated with suicidality.

Data regarding any direct correlation between the microbiota-gut-brain axis and suicidal behaviour or ideation is limited. Figure 1 presents the discovered puzzle pieces of the association among the gastrointestinal microbiome, the inflammatory system, and self-injury, including suicidality.

### Gut microbiome

Non-suicidal self-injury (NSSI), which is defined as a repetitive, direct, and unacceptable injury to the body of one’s own but without any suicidal ideation, is considerably important (Lloyd-Richardson et al. 2007). The global NSSI prevalence occurring in school students for at least one time is 17.2% (Swannell et al. 2014). Also, NSSI can increase the risk of suicide significantly (Ribeiro et al. 2016). The differences in gut-related microbiota between subjects with NSSI, depression cases without NSSI, and healthy controls were assessed in the study by Cai et al. (Cai et al. 2022) They showed significant differences regarding gut microbiota diversity between the subjects with NSSI and healthy controls. In addition, the species uniformity index of NSSI patients was significantly reduced, compared with normal subjects, based on the Shannon and Simpson indices. In the study by Maes et al. (Maes et al. 2023), it was shown that six gut microbiomes, including Clostridium, Butyricicoccus, Desulfovibrio piger, and Parabacteroides merdae demonstrated inverse associations, but Fusicatenibacter and Hungatella demonstrated positive association and correlated with suicidal behaviours in patients with adverse childhood experience. In the study by Ahrens et al. (Ahrens et al. 2022), it was shown that salivary bacteria are also associated with suicidal ideation. They reported that the saliva microbiome was dominated by Veillonella in subjects with suicidal ideation. Also, Megasphaera was significantly higher in patients with suicidal ideation.

On the other hand, Mendoza-Larios et al. (Mendoza-Larios et al. 2021) investigated the link between Toxoplasma gondii seropositivity and suicide by analysing serum samples from 89 suicide cases and 58 non-suicide controls. Anti-Toxoplasma gondii IgG antibodies were detected in 9.0% of cases and 10.3% of controls, with no significant association. IgM antibodies were absent in both groups except for one control. Antibody levels were similar across sex and age groups. The findings do not support a connection between Toxoplasma gondii seropositivity and suicide, though low statistical power suggests further research is needed to confirm these results.

### Intestinal secretion

Zonulin, first described in 2000 (Fasano et al. 2000), is a protein that modulates the small intestine permeability by inducing the disassembly of cellular tight junctions in the small intestine and duodenum, resulting in increased permeability (Fasano 2011). On the other hand, intestinal fatty acid binding protein (I-FABP), which is a cytoplasmic protein, is found in small intestine enterocytes. Elevated I-FABP levels are correlated with enterocyte damage (Adriaanse et al. 2013; Piton et al. 2015). Ohlsson et al. (Ohlsson et al. 2019) showed higher I-FABP levels in patients with recent suicide attempts than in patients with major depressive disorder (MDD) without any history of suicide attempts or healthy controls. Also, they revealed lower zonulin levels in subjects with recent suicide attempts. Moreover, the patients with recent suicidal attempts continued to have significantly lower zonulin and higher I-FABP levels compared with healthy controls or patients with MDD without any history of suicide attempts. In addition, interleukin-6 (IL-6) correlated positively with I-FABP levels and negatively with zonulin levels (Ohlsson et al. 2019).

### Cytokines

There is scarce available data regarding the underlying pathobiology behind inflammation in depression and suicidal behaviour. In a preprint article by Almulla et al. (Almulla et al. 2023), the effects of growth factors, chemokines, cytokines, T helper (Th) phenotypes, macrophages, immune-inflammatory response system, neuro-immunotoxicity, and compensatory immunoregulatory system profiles in subjects in the first episode and acute stage of major dysmood disorder with no prior history of psychiatric disorders were evaluated and compared with healthy controls. They showed that suicidal behaviour in the first episode of major dysmood disorder is positively associated with platelet-derived growth factor, interleukin (IL)-16, and M1 macrophages. Also, it is negatively associated with soluble IL receptor antagonists. Moreover, experimental and clinical evidence reveal that gut microbiota has an important role in the gut-brain axis, interacting not only locally but also directly with CNS through metabolic and neuroendocrine pathways. The microbiota-gut-brain axis, which links gastrointestinal function with cognitive and emotional brain centres, is suggested to be related to psychiatric disorders (Carabotti et al. 2015).

The contribution of inflammatory changes to symptoms in suicidal patients is not clear. However, there have been case report studies that described the association between suicidal behaviour and immune-modulating interferon (IFN) therapies, suggesting a causal relationship (Rifflet et al. 1998; Nickel et al. 2005). Animal studies have proposed that administering IL-6 and IL-1β increases the serotonin metabolite 5-hydroxyindoleacetic acid in various brain regions (Dunn 1992; Wang and Dunn 1998). On the other hand, altered levels of monoamine metabolites in cerebrospinal fluid have been observed in suicide attempters (Mann 2003). It has been reported by Lindqvist et al. (Lindqvist et al. 2009) that IL-6 levels in cerebrospinal fluid were significantly higher in subjects who attempted suicide than in healthy controls. Also, they revealed that violent suicide attempters had the highest IL-6 levels.

### Laxative abuse

There is a theory indicating that laxative abuse might change gut-brain signalling. In a study by Lengvenyte et al. (Lengvenyte et al. 2022), a group of patients with eating disorders was collected and divided into two groups, including patients with lifetime laxative misuse and non-misusers. The results revealed that patients with lifetime laxative misuse were more likely to attempt suicide based on their history. Also, the patients with suicidal attempts in the last 28 days reported more days of laxative misuse. Moreover, it was reported that laxative use days in the past 28 days was also associated with suicidal ideation.

## Microbiome-inflammation roles in mental disorders leading to suicide

**Figure 2.**
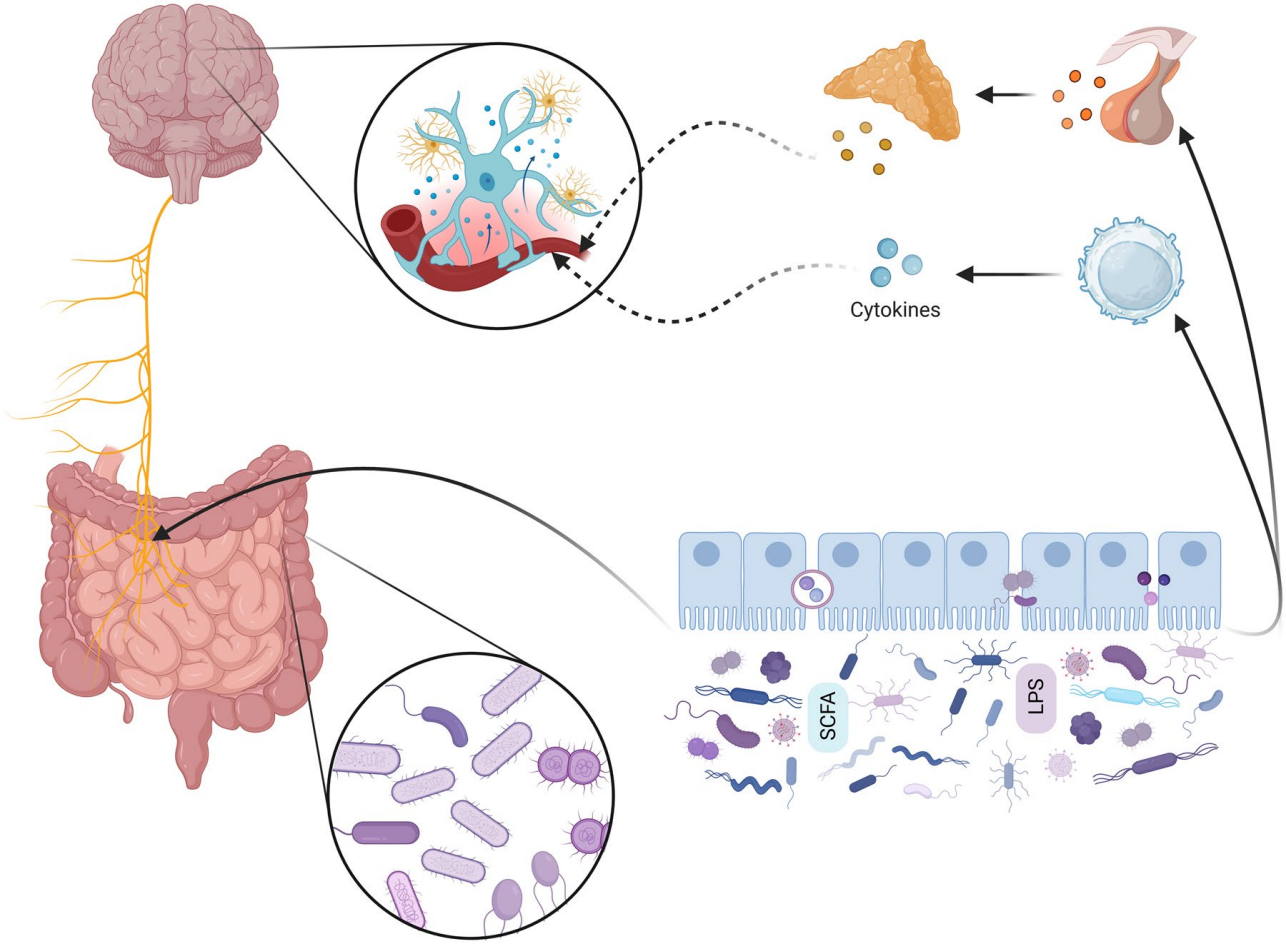
The gastrointestinal microbiome can decrease short-chain fatty acid (SCFA), secrete lipopolysaccharides (LPS), and increase intestinal wall permeability (“dysbiosis”), which leads to the activation of toll-like receptor 4, the circulation of extracellular vesicles, and the circulation of neurotransmitters. Thus, dysbiosis, by activating the vagus nerve, increasing the secretion of hormones of the hypothalamic-pituitary-adrenal axis, and inducing the production of inflammatory cytokines from T cells, causes disruption of microglial activity, astrocyte atrophy, changes in brain function, and mental disorders.

It is estimated that nearly 90% of suicides occur in individuals suffering from mental disorders. Among these, MDD is associated with the highest rate, and a half to two-thirds of reported suicide cases are considered to be among MDD patients (Olgiati and Serretti 2023). Substance use disorder (SUD), generalised anxiety disorder (GAD), and psychosis are among the other most prominent risk factors that have shown a high prevalence of suicide attempts in most cases (Goldston et al. 2009; Bachmann 2018). Figure 2 shows the development of mental disorders by the gut microbiome through nerves, hormones, and inflammation.

### Major depressive disorder

Accumulating evidence suggests that the most prominent phyla demonstrating incremental patterns in MDD patients compared to healthy individuals are Proteobacteria, Bacteroidetes, and Actinobacteria. Meanwhile, lower Firmicutes are reported in the bacterial species of the gut among MDD patients. In terms of genus changes, genera including Enterobacteriaceae, Eubacterium, Bilophila, Allistipes, Clostridium Parabacteroides, Anaerostipes, Streptococcus, Blautia, Klebsiella, Lachnospiraceae incertae sedis, Phascolarctobacterium, Parasutterella, and Lactobacillus spp. are shown to be higher in MDD. The genera with lower existence in MDD patients are Faecalibacterium, Dialister, Bifidobacterium, Ruminococcus, and Escherichia/Shigella (Cheung et al. 2019; Yang et al. 2020; Caso et al. 2021; Foster et al. 2021).

Segmented filamentous bacteria related to the Clostridium genus are also known to have a role in increasing depression susceptibility by promoting serum amyloid protein production by the host, which in turn results in the stimulation of the Th17 cell production, known to have a role in depression occurrence (Beurel et al. 2013; Medina-Rodriguez et al. 2020). In a study analysing the depression microbiota, a significant increase in the tumour necrosis factor (TNF)-α, IFN-γ, IL-6, and IL-1 levels was evident, indicating a prominent role for inflammation in the relationship between gut microbiota and the pathogenesis of MDD (Liu et al. 2020).

Some gut bacteria are shown to affect the inflammatory process directly. Bifidobacterium spp, as an example, is able to suppress inflammation by inhibiting the nuclear factor-κB pathway. The lower abundance of this genus is also evident in MDD, confirming this hypothesis. Lactobacillus spp., with higher abundance in MDD patients, is reported to be involved in stimulating macrophages to produce caspase-1-dependent IL-1β, resulting in inflammasome activation. Some members of the Lachnospiraceae family are also reported to induce Foxp3 regulatory T cells and suppress IL-1β and IL-6 (Wong et al. 2016).

Firmicutes are among the gut bacterial species that can ferment carbohydrates to short-chain fatty acids (SCFAs). The lack of SCFA might lead to intestinal barrier dysfunction, called dysbiosis, causing the pathogens and their metabolites to stimulate the host’s immune responses. Furthermore, SCFAs are responsible for the activation of regulatory T cells in addition to the inhibition of pro-inflammatory cytokine production. With all the above-mentioned mechanisms, Firmicutes are inflammatory response suppressors. Considering a close correlation between depression and inflammation or cell-mediated immunity, it is not surprising to see lower firmicutes in MDD patients (Dowlati et al. 2010; Leonard and Maes 2012; Huang et al. 2018).

Among the most prominent mechanisms contributing to MDD, dysbiosis has attracted lots of attention. This usually occurs due to changes in the gut microbiome caused by many factors, including stress, resulting in the production of inflammatory cytokines. It can also alter the blood-brain barrier (BBB) permeability as well, increasing brain cytokines and neuroinflammation. Microglial activation and astrocyte atrophy occur as a result, affecting various brain networks encompassing mood regulation, memory, emotions, and learning. These mechanisms can eventually lead to MDD or anxiety. It also noted that inflammatory cytokines such as IL-6 and IFN-γ can shift the synthesis of serotonin to kynurenine and its metabolites through the indoleamine-2,3-dioxygenase (IDO) synthesis, which alters the tryptophan metabolism. The Kynurenine pathway is considered prominent in the pathogenesis of MDD and psychiatric disorders (Carlessi et al. 2021). It is proposed to occur mainly for Alistipes (Caso et al. 2021).

Furthermore, the effect of the gut microbiota on immunity is said to occur directly through the activation of the vagus nerve (Rogers et al. 2016). Meanwhile, the vagus nerve can control the immune responses through the cholinergic anti‐inflammatory pathway and the hypothalamic-pituitary-adrenal (HPA) axis activation following high inflammatory cytokine conditions (Peirce and Alviña 2019).

Interestingly, another hypothesis for MDD pathogenesis asserts that bacterial translocation-induced inflammation can cause MDD through oxidative/nitrosative stress. Besides, the lipopolysaccharides (LPS) of Gram-negative bacteria like Alistipes and Bilophila can stimulate innate immunity by activating toll-like receptor 4 (TLR-4). The activation of TLR-4 is known to be associated with depressive-like behaviours (Caso et al. 2021).

It is also noted that five metabolites from the gut microbiota related to inflammation were considered to be involved in MDD as follows: LysoPC(16:0), LysoPC(20:0), Deoxycholic acid, Taurocholic acid, and Docosahexaenoic acid (Bai et al. 2021). The pathogenesis of depression is also influenced by non-bacterial microbiota. By promoting the diversity of intestinal bacteria genotypes and phenotypes, Bacteriophages are beneficial partners for bacterial microbiota (Shkoporov et al. 2022). There has been evidence for shifts in bacteriophage composition in patients with MDD, particularly changes in Caudovirales (Yang et al. 2020). Administering Bifidobacterium longum NK46 and Lactobacillus mucosae NK41 probiotic strains can inhibit the activation of TNF-α and nuclear factor kappa B (NF-B), which are both components involved in the pathogenicity of depression (Sang-Kap 2019).

Some markers of structural barrier integrity and paracellular permeability, such as zonulin and I-FABP, assess gastrointestinal permeability in MDD. One study reported significantly higher levels of zonulin and I-FABP in MDD patients than in controls (Stevens et al. 2018). Alvarez-Mon et al. also noted a substantial increase in I-FABP in patients with MDD compared to controls (Alvarez-Mon et al. 2019). In another study, patients with different psychiatric disorders who had experienced recent suicidal ideation had higher levels of I-FABP but lower levels of zonulin compared to both MDD patients without a history of suicide attempts and healthy controls (Ohlsson et al. 2019).

### Psychosis and schizophrenia

In patients with schizophrenia, the gut microbiota exhibits significant alterations compared to healthy individuals. It has been demonstrated that alterations in the following phyla are observed in patients at risk or with schizophrenia: Actinobacteria, Proteobacteria, Bacteroidetes, Fusobacteria, and Firmicutes (Kraeuter et al. 2020). At the genus level, abundance differences are observed among schizophrenia patients, of which higher Prevotella, Escherichia/Shigella, Megasphaera, Eggerthella, Lactobacillus, Succinivibrio, Acidaminococcus, Collinsella, Eubacterium, Mogibacterium, Desulfovibrio, Bulleidia, Corynebacterium, Atopobium, and Veillonella, in addition to lower Bacteroides, Lachnospiraceae, Haemophilus, Roseburia, Coprococcus, Faecalibacterium, Adlercreutzia, Anaerostipes, Turicibacter, and Streptococcus, were the most prominent (Li et al. 2020; 2021; McGuinness et al. 2022; Li et al. 2023; 2024).

Although evidence for Ruminococcaceae has been mixed, the evidence suggests the correlation of Ruminococcaceae with reduced severity of psychotic symptoms, specifically negative ones, and particularly improved self-reported physical health (Nguyen et al. 2021). In a study by Zheng et al. Veillonellaceae OTU191 was negatively associated with Positive and Negative Syndrome Scale (PANNS) scores, whereas Bacteroidaceae OTU172, Streptococcaceae OTU834 and two Lachnospiraceae OTUs (477 and 629) were positively associated with PANSS scores (Zheng et al. 2019). Moreover, Succinivibrio was found to be positively correlated with total and general PANSS scores, and Corynebacterium was negatively associated with the negative symptom scores of the PANSS (Li et al. 2020). Furthermore, the utilisation of a probiotic supplement containing Lactobacilli and Bifidobacterium bifidum (along with vitamin D) in schizophrenic subjects is shown to cause a significant improvement in general and total PANSS scores (Ghaderi et al. 2019).

Butyrate-producing bacteria like Coprococcus and Roseburia, with lower levels in schizophrenia, produce butyrate and other SCFAs *via* anaerobic fermentation of diet fibres known to have many benefits by intestinal hemostasis and affecting the immune system. The lower levels of SCFAs in schizophrenia can have many impacts, like that described in MDD, through systemic inflammation (McGuinness et al. 2022). These dysbiotic alterations can affect CNS functions *via* inflammation and changes in spinal and vagal nerve pathways (Rogers et al. 2016). Besides, metabolic pathways related to inflammatory cytokines are shown to be altered in schizophrenia patients. Kdo2-lipid A biosynthesis and trimethylamine-N-oxide reductase are among those pathways; their alterations result in a lasting pro-inflammatory state in schizophrenia patients (Nguyen et al. 2021).

From another perspective, inflammation is associated with an imbalance in the oxidation/antioxidant system, leading to increased consumption of superoxide dismutase (Tan et al. 2015). In a study, its levels were inversely correlated with the presence of Eubacterium, Collinsella, Lactobacillus, Corynebacterium, Bulleidia, Mogibacterium, and Succinivibrio, but positively correlated with Faecalibacterium, Ruminococcus, and cognition scores (Li et al. 2024).

Fond et al. performed a study on latent toxoplasma infection in real-world schizophrenia. This study found a high prevalence (73.6%) of latent Toxoplasma gondii infection in a national sample. Infection was significantly linked to higher negative and excitement symptoms in schizophrenia, particularly reference delusions and alogia, as well as chronic low-grade inflammation and extrapyramidal symptoms. However, no associations were found with age, gender, schizophrenia onset, suicide behaviour, or cognitive deficits. Toxoplasma antibody-associated traits were linked to lower depressive symptoms and reduced chronic inflammation but did not correlate with cognitive scores. These findings suggest a potential role of Toxoplasma gondii in schizophrenia symptomatology and immune response, warranting further research (Fond et al. 2018). Also, Helicobacter pylori infection was mentioned as potentially influencing schizophrenia symptoms by causing dopaminergic dysfunction and inflammation (Zajkowska et al. 2024). Additionally, in a study by Li et al. increased serum homocysteine levels, which promote inflammatory response, were positively associated with Eubacterium, Lactobacillus, Corynebacterium, Mogibacterium, and Bulleidia and decreased cognitive function in patients with schizophrenia (Li et al. 2023).

Considering the effect of gut microbiota on immune system regulation, bacterial metabolites can trigger innate and adaptive immunity. Complement C1q and the major histocompatibility complex 1 (MHC1) are important components of the immune system that have been shown to have roles in brain synaptogenesis as well as synaptic pruning. These events might result in the pathogenesis of developmental brain disorders, including schizophrenia. High levels of immune complexes with C1q in schizophrenia patients compared to healthy individuals can confirm this hypothesis (Severance et al. 2012; 2015).

### Anxiety disorders

Higher abundances of Paraprevotella, Caldivirga, Euryarchaeota, Desulfovibrionales, and Porphyro­monadaceae, as well as lower abundances of Faecalibacterium, Eubacterium rectale, Butyricicoccus, Lachnospira, Sutterella, Vagococcus, Lactobacillus, Barnesiella, and Paludibacter, are reported in GAD patients compared to healthy controls (Jiang et al. 2018; Cheng et al. 2022). It is also demonstrated that the gut microbiota generates reduced levels of neurotoxic metabolites after administration of Bacteroides fragilis, including 4-EPS, serum glycolate, and imidazole propionate, improving gut permeability and reducing anxiety-like behaviour (Bai et al. 2021).

Endotoxemia is a condition that leads to immune response activation due to the entrance of gut bacterial endotoxins following dysbiosis. Stress and anxiety are found to be prominent initiating factors for the mentioned mechanism. Subsequently, the peripheral inflammation can reach the brain and cause neuroinflammation through various mechanisms encompassing (i) passage of cytokines *via* BBB, (ii) migration of activated immune cells to the brain, and (iii) transmission of inflammatory signals to the brain by afferent nerves from peripheral cytokines as well as pathogen‐associated molecular patterns. Eventually, the microglia become activated and release reactive oxygen and nitrogen species, which can cause neural toxicity. Besides, dysfunction in the synthesis of monoamine neurotransmitters encompassing dopamine, serotonin, and norepinephrine occurs as a result. Finally, these events lead to improper brain activity and mental disorders like anxiety (Peirce and Alviña 2019).

### Substance use disorder

Decreased relative abundance of Firmicutes, mainly Lactobacilli and Enterococci, as well as Actinobacteria, mainly Bifidobacteria, is reported to occur in alcohol users. A decrease in gut Firmicutes is also evident in morphine and cocaine users. Conversely, an increased relative abundance of Bacteroidetes is seen in all groups (Meckel and Kiraly 2019). Opioid users demonstrate higher Bifidobacterium and lower Bacteroidacea, Clostridiales XIV, and Ruminococcaceae (Barengolts et al. 2018; Meckel and Kiraly 2019).

Among individuals with methamphetamine use disorder (MUD), Collinsella, a genus from the Coriobacteriaceae family, and Actinobacteria phylum are highly associated with MUD. This bacterium induces the production of IL-17A from epithelial cells, which eventually results in lower expression of ZO-1 protein, contributing to gut hyperpermeability, which is shown to be involved in inflammatory status. Another bacterium with anti-inflammatory features, which shows reduced levels in MUD patients, resulting in the inflammatory status in MUD, is Faecalibacterium. The anti-inflammatory effect of this gut bacteria is implemented through inhibiting NF-κB and inducing IL-10. Considering the anti-inflammatory properties of IL-10, other bacteria from the Firmicutes phylum, Lactobacillales, with decreased levels in MUD patients, have shown positive correlations with IL-10 levels; therefore, the evidence indicated that these can modulate cytokine release (Iyer and Cheng 2012; Vemuri et al. 2017; Deng et al. 2021).

It is also demonstrated that alcohol use can increase gut permeability *via* increasing LPS and peptidoglycan produced by gut microbiota. As mentioned earlier, permeability alterations can cause gut bacterial translocation into the circulation, eventually inducing inflammatory status and release of inflammatory cytokines, which can cross the BBB and alter the behavioural responses of the brain to drugs (Kraeuter et al. 2020; Li et al. 2021). The dysbiosis also occurs in other SUDs, increasing the permeability of the gut and resulting in systemic inflammation that, in turn, can change the microglial activity. Gut bacterial metabolites and the disrupted homeostasis of the gut can affect the vagus nerve and the HPA axis and impact the brain downstream and hormone release, respectively (Nguyen et al. 2021).

## Discussion

This review demonstrated the functional roles of the bacterial and non-bacterial microbiome of the gastrointestinal tract in suicide and the most prominent mental disorders *via* the inflammatory system, although some pieces of the puzzle are still unresolved. The changes in the balance of gut microbiota influence the CNS environment from non-inflammatory to inflammatory, which is caused by an influx of pro-inflammatory cytokines into the brain. As a consequence, gut microbiota imbalances may be associated with mental disorders, self-injury, or suicide. The intestinal microbiota may also be disrupted by lifestyle disruption due to psychiatric disorders or constipation caused by the anticholinergic effects of antipsychotic and antidepressant medications.

Emerging evidence indicates that gut microbiota influences brain function and behaviour through multiple mechanisms, including the production of neurotransmitters, modulation of immune responses, and regulation of the stress response system (Peirce and Alviña 2019). Specific bacterial genera contribute to the synthesis of neuroactive compounds such as serotonin, dopamine, and γ-aminobutyric acid, which are critical in mood regulation (Yang et al. 2020). For example, Lactobacillus and Bifidobacterium are involved in γ-aminobutyric acid production, which reduces neuronal excitability and is associated with anxiety and depression (Huang et al. 2018). Additionally, gut bacteria influence the metabolism of tryptophan, a precursor of serotonin. Disruptions in this pathway, such as increased conversion of tryptophan to kynurenine due to an inflammatory response, may lead to reduced serotonin levels in the brain, which has been implicated in suicidal thoughts and behaviours (Carlessi et al. 2021; Caso et al. 2021).

Our findings suggest that the association between certain microbiota genera and self-harming behaviour may be mediated through these pathways. For instance, the increased presence of Escherichia-Shigella, a genus known to trigger inflammatory responses, may lead to elevated levels of pro-inflammatory cytokines such as IL-6 and TNF-α (Sang-Kap 2019; Liu et al. 2020). These cytokines can activate microglial cells in the brain, leading to neuroinflammation and alterations in neurotransmitter systems that regulate mood and impulse control (Sang-Kap 2019; Liu et al. 2020). Conversely, the decreased abundance of Faecalibacterium, a genus with anti-inflammatory properties, might result in a lower production of butyrate, an SCFA that plays a protective role in maintaining the integrity of the BBB and reducing neuroinflammation (Dowlati et al. 2010; Leonard and Maes 2012; Huang et al. 2018). These mechanisms provide a potential link between gut microbiota alterations and the biological processes underlying self-harming behaviour. Further studies incorporating functional analyses of microbial metabolites and their interactions with neural pathways are necessary to establish causal relationships in this complex interplay.

Overall, while distinct psychiatric disorders exhibit unique microbiota alterations, there are notable commonalities and differences across conditions. Depression is frequently associated with decreased beneficial bacteria such as Faecalibacterium and Bifidobacterium and increased pro-inflammatory taxa like Enterobacteriaceae. Schizophrenia shows reductions in Blautia and Akkermansia, alongside increased Lactobacillus, which may influence neurotransmitter metabolism. Anxiety disorders often mirror depressive microbiota patterns, with lower Lactobacillus and Bifidobacterium levels, indicating a shared gut-brain axis disruption. In substance use disorders, dysbiosis manifests with a decrease in SCFA-producing bacteria and an overrepresentation of pro-inflammatory species, reflecting a potential link between gut inflammation and addiction pathways. Despite these disorder-specific changes, common themes emerge, including a reduction in anti-inflammatory, SCFA-producing bacteria and an increase in pro-inflammatory taxa, suggesting a broader gut dysbiosis pattern across psychiatric illnesses. Understanding these shared and unique microbial signatures could pave the way for targeted microbiome-based interventions in mental health.

Regarding the treatment strategies, Cui et al. (Cui et al. 2025) conducted research regarding the application of psychobiotics for the treatment of mental disorders. They revealed that psychobiotics, a specialised class of probiotics, positively influence mental health by modulating gut-brain communication. Key bacteria include Lactobacillus, Streptococcus, and Bifidobacterium. Unlike traditional probiotics, psychobiotics can produce or stimulate neurotransmitters, SCFAs, gut hormones, and anti-inflammatory cytokines, impacting central nervous system signalling. Their use has grown over the past decade for treating mental disorders, including depression, anxiety, and neurodevelopmental diseases. Bacillus licheniformis shows potential as an adjunct therapy for depression. However, evidence remains limited, and further research is needed to confirm their clinical efficacy and mechanisms in managing mental and neurological disorders.

It has been demonstrated that higher IL-6 in cerebrospinal fluid, higher I-FABP, and lower zonulin levels are associated with suicidal attempts. In addition, it was revealed that the species uniformity index in NSSI patients is significantly reduced. In terms of the gut microbiome, Clostridium, Butyricicoccus, Desulfovibrio piger, and Parabacteroides merdae have inverse associations with suicidal behaviours or ideation, while Fusicatenibacter, Hungatella, Veillonella, and Megasphaera have a positive association with suicidal behaviours or ideation. Furthermore, laxative abuse in patients with eating disorders has been strongly correlated with suicidal attempts (Table S1).

Alterations in the gut microbiome are reported in various mental disorders contributing to suicide risk. The most prominent and prevalent risk factors of suicide attempts, encompassing MDD, psychosis, and GAD among mental disorders and SUD, were investigated, revealing that Bacteroidetes and Firmicutes are among the gut bacteria that show prominent alterations in most of these disorders. Besides, alterations in the gut bacteria and dysbiosis leading to increased gut permeability can, in turn, result in inflammatory status. The pro-inflammatory cytokines can reach the brain and pass the BBB, eventually influencing the microglial activity by neuroinflammation, leading to the progression of these disorders as suicide risk factors. Meanwhile, some other mechanisms are known to have a role in developing these disorders through the inflammatory process, of which, effects of disrupted gut homeostasis on the vagus nerve and HPA axis, in addition to the Kynurenine pathway, have attracted lots of attention (Table S2). It is worth noting that the direct effect of some gut bacteria on the immune system is hypothesised; however, more investigations are needed to help us draw a firm conclusion.

In conclusion, changes in the balance of gut microbiota may influence the central nervous system environment from non-inflammatory to inflammatory caused by an influx of pro-inflammatory cytokines into the brain. Consequently, gut microbiota imbalances may be associated with mental disorders, self-injury, or suicide. Although the gut microbiome has been linked to psychiatric disorders, its role in suicidal behaviour is not yet well understood. Existing evidence suggests a potential association between microbiome alterations and suicidality, but further research is needed to define these relationships and underlying mechanisms better. Other contributing factors, including genetic predisposition, environmental influences, and neurochemical pathways, should be considered.

## Supplementary Material

Supplemental Material

## Data Availability

Not applicable.

## References

[CIT0001] Adriaanse MPM et al. 2013. Serum I‐FABP as marker for enterocyte damage in coeliac disease and its relation to villous atrophy and circulating autoantibodies. Aliment Pharmacol Ther. 37(4):482–490. 10.1111/apt.12194 23289539

[CIT0002] Ahrens AP et al. 2022. Saliva microbiome, dietary, and genetic markers are associated with suicidal ideation in university students. Sci Rep. 12(1):14306. 10.1038/s41598-022-18020-2 35995968 PMC9395396

[CIT0003] Almulla AF, Abo Algon AA, Tunvirachaisakul C, Al-Hakeim HK, Maes MT. 2023. helper-1 activation via interleukin-16 is a key phenomenon in the acute phase of severe, first-episode major depressive disorder and suicidal behaviors. medRxiv. 2023.04.16.23288643. 10.1016/j.jare.2023.11.012 PMC11464466 37967811

[CIT0004] Alvarez-Mon MA et al. 2019. Abnormal distribution and function of circulating monocytes and enhanced bacterial translocation in major depressive disorder. Front Psychiatry. 10:812. 10.3389/fpsyt.2019.00812 31803077 PMC6873610

[CIT0005] Bachmann S. 2018. Epidemiology of Suicide and the Psychiatric Perspective. Int J Environ Res Public Health. 15(7):1425. 10.3390/ijerph15071425 29986446 PMC6068947

[CIT0006] Baethge C, Goldbeck-Wood S, Mertens S. 2019. SANRA—a scale for the quality assessment of narrative review articles. Res Integr Peer Rev. 4(1):5. 10.1186/s41073-019-0064-8 30962953 PMC6434870

[CIT0007] Bai S et al. 2021. Gut microbiota-derived inflammation-related serum metabolites as potential biomarkers for major depressive disorder. J Inflamm Res. 14:3755–3766. 10.2147/JIR.S324922 34393496 PMC8354734

[CIT0008] Barengolts E et al. 2018. Gut microbiota varies by opioid use, circulating leptin and oxytocin in African American men with diabetes and high burden of chronic disease. PLoS One. 13(3):e0194171. 10.1371/journal.pone.0194171 29596446 PMC5875756

[CIT0009] Beurel E, Harrington LE, Jope RS. 2013. Inflammatory T helper 17 cells promote depression-like behavior in mice. Biol Psychiatry. 73(7):622–630. 10.1016/j.biopsych.2012.09.021 23174342 PMC3582833

[CIT0010] Cai LF et al. 2022. Association between non-suicidal self-injury and gut microbial characteristics in chinese adolescent. Neuropsychiatr Dis Treat. 18:1315–1328. 10.2147/NDT.S360588 35799798 PMC9255420

[CIT0011] Carabotti M, Scirocco A, Maselli MA, Severi C. 2015. The gut-brain axis: interactions between enteric microbiota, central and enteric nervous systems. Ann Gastroenterol. 28(2):203–209. 25830558 PMC4367209

[CIT0012] Carlessi AS, Borba LA, Zugno AI, Quevedo J, Réus GZ. 2021. Gut microbiota–brain axis in depression: the role of neuroinflammation. Eur J Neurosci. 53(1):222–235. 10.1111/ejn.14631 31785168

[CIT0013] Caso JR et al. 2021. Gut microbiota, innate immune pathways, and inflammatory control mechanisms in patients with major depressive disorder. Transl Psychiatry. 11(1):645. 10.1038/s41398-021-01755-3 34934041 PMC8692500

[CIT0014] Cerel J et al. 2019. How many people are exposed to suicide? Not six. *Suicide and* Suicide Life Threat Behav. 49(2):529–534. 10.1111/sltb.12450 29512876

[CIT0015] Chang B, Gitlin D, Patel R. 2011. The depressed patient and suicidal patient in the emergency department: evidence-based management and treatment strategies. Emerg Med Pract. 13(9):1–23. quiz 10.2310/em.4373 22164363

[CIT0016] Cheng Y et al. 2022. Relationship between intestinal flora, inflammation, BDNF gene polymorphism and generalized anxiety disorder: a clinical investigation. Medicine (Baltimore). 101(29):e28910. 10.1097/MD.0000000000028910 35866837 PMC9302347

[CIT0017] Cheung SG et al. 2019. Systematic review of gut microbiota and major depression. Front Psychiatry. 10:34. 10.3389/fpsyt.2019.00034 30804820 PMC6378305

[CIT0018] Cui J et al. 2025. Application of psychobiotics in clinical treatment of mental disorders: neurodevelopmental disorders, neurodegenerative diseases, depression and anxiety. Interdiscip Med. 3(1). 10.1002/INMD.20240041

[CIT0019] Deng D et al. 2021. Altered fecal microbiota correlated with systemic inflammation in male subjects with methamphetamine use disorder. Front Cell Infect Microbiol. 11:783917. 10.3389/fcimb.2021.783917 34869080 PMC8637621

[CIT0020] Dowlati Y et al. 2010. A meta-analysis of cytokines in major depression. Biol Psychiatry. 67(5):446–457. 10.1016/j.biopsych.2009.09.033 20015486

[CIT0021] Dunn AJ. 1992. Endotoxin-induced activation of cerebral catecholamine and serotonin metabolism: comparison with interleukin-1. J Pharmacol Exp Ther. 261(3):964–969. 10.1016/S0022-3565(25)11171-3 1602402

[CIT0022] Eisenstein M. 2016. Microbiome: bacterial broadband. Nature. 533(7603):S104–S6. 10.1038/533S104a 27191486

[CIT0023] Fasano A et al. 2000. Zonulin, a newly discovered modulator of intestinal permeability, and its expression in coeliac disease. Lancet. 355(9214):1518–1519. 10.1016/S0140-6736(00)02169-3 10801176

[CIT0024] Fasano A. 2011. Zonulin and its regulation of intestinal barrier function: the biological door to inflammation, autoimmunity, and cancer. Physiol Rev. 91(1):151–175. 10.1152/physrev.00003.2008 21248165

[CIT0025] Fond G et al. 2018. Latent toxoplasma infection in real-world schizophrenia: results from the national FACE-SZ cohort. Schizophr Res. 201:373–380. 10.1016/j.schres.2018.05.007 29843964

[CIT0026] Foster JA, Baker GB, Dursun SM. 2021. The relationship between the gut microbiome-immune system-brain axis and major depressive disorder. Front Neurol. 12:721126. 10.3389/fneur.2021.721126 34650506 PMC8508781

[CIT0027] Ghaderi A et al. 2019. Clinical and metabolic response to vitamin D plus probiotic in schizophrenia patients. BMC Psychiatry. 19(1):77. 10.1186/s12888-019-2059-x 30791895 PMC6383260

[CIT0028] Goldston DB et al. 2009. Psychiatric diagnoses as contemporaneous risk factors for suicide attempts among adolescents and young adults: developmental changes. J Consult Clin Psychol. 77(2):281–290. 10.1037/a0014732 19309187 PMC2819300

[CIT0029] Guo Z et al. 2024. Disruption of the gut microbiota-inflammation-brain axis in unmedicated bipolar disorder II depression. Transl Psychiatry. 14(1):495. 10.1038/s41398-024-03207-0 39695130 PMC11655654

[CIT0030] Huang Y et al. 2018. Possible association of Firmicutes in the gut microbiota of patients with major depressive disorder. Neuropsychiatr Dis Treat. 14:3329–3337. 10.2147/NDT.S188340 30584306 PMC6284853

[CIT0031] Iyer SS, Cheng G. 2012. Role of interleukin 10 transcriptional regulation in inflammation and autoimmune disease. Crit Rev Immunol. 32(1):23–63. 10.1615/critrevimmunol.v32.i1.30 22428854 PMC3410706

[CIT0032] Järbrink-Sehgal E, Andreasson A. 2020. The gut microbiota and mental health in adults. Curr Opin Neurobiol. 62:102–114. 10.1016/j.conb.2020.01.016 32163822

[CIT0033] Jiang H-Y et al. 2018. Altered gut microbiota profile in patients with generalized anxiety disorder. J Psychiatr Res. 104:130–136. 10.1016/j.jpsychires.2018.07.007 30029052

[CIT0034] Khan MR et al. 2024. Gut-brain axis: exploring the link between digestive health and mental health. Indus J Biosci Res. 2(02):1307–1313.

[CIT0035] Kraeuter A-K, Phillips R, Sarnyai Z. 2020. The gut microbiome in psychosis from mice to men: a systematic review of preclinical and clinical studies. Front Psychiatry. 11:799. 10.3389/fpsyt.2020.00799 32903683 PMC7438757

[CIT0036] Lengvenyte A et al. 2022. A specific association between laxative misuse and suicidal behaviours in patients with anorexia nervosa and bulimia nervosa. Eat Weight Disord. 27(1):307–315. 10.1007/s40519-021-01180-x 33797033

[CIT0037] Leonard B, Maes M. 2012. Mechanistic explanations how cell-mediated immune activation, inflammation and oxidative and nitrosative stress pathways and their sequels and concomitants play a role in the pathophysiology of unipolar depression. Neurosci Biobehav Rev. 36(2):764–785. 10.1016/j.neubiorev.2011.12.005 22197082

[CIT0038] Li H et al. 2023. Association of serum homocysteine levels with intestinal flora and cognitive function in schizophrenia. J Psychiatr Res. 159:258–265. 10.1016/j.jpsychires.2023.01.045 36773527

[CIT0039] Li H et al. 2024. The relationship between the gut microbiota and oxidative stress in the cognitive function of schizophrenia: a pilot study in China. Schizophr Res. 267:444–450. 10.1016/j.schres.2024.03.053 38643725

[CIT0040] Li S et al. 2020. Altered gut microbiota associated with symptom severity in schizophrenia. PeerJ. 8:e9574. 10.7717/peerj.9574 32821537 PMC7395597

[CIT0041] Li S et al. 2021. The gut microbiome is associated with brain structure and function in schizophrenia. Sci Rep. 11(1):9743. 10.1038/s41598-021-89166-8 33963227 PMC8105323

[CIT0042] Lindqvist D et al. 2009. Interleukin-6 is elevated in the cerebrospinal fluid of suicide attempters and related to symptom severity. Biol Psychiatry. 66(3):287–292. 10.1016/j.biopsych.2009.01.030 19268915

[CIT0043] Liu S et al. 2020. Gut microbiota regulates depression-like behavior in rats through the neuroendocrine-immune-mitochondrial pathway. Neuropsychiatr Dis Treat. 16:859–869. 10.2147/NDT.S243551 32280227 PMC7127849

[CIT0044] Lloyd-Richardson EE, Perrine N, Dierker L, Kelley ML. 2007. Characteristics and functions of non-suicidal self-injury in a community sample of adolescents. Psychol Med. 37(8):1183–1192. 10.1017/S003329170700027X 17349105 PMC2538378

[CIT0045] Maes M et al. 2023. Adverse childhood experiences and reoccurrence of illness impact the gut microbiome, which affects suicidal behaviours and the phenome of major depression: towards enterotypic phenotypes. Acta Neuropsychiatr. 35(6):328–345. 10.1017/neu.2023.21 37052305

[CIT0046] Mann JJ. 2003. Neurobiology of suicidal behaviour. Nat Rev Neurosci. 4(10):819–828. 10.1038/nrn1220 14523381

[CIT0047] McGuinness AJ et al. 2022. A systematic review of gut microbiota composition in observational studies of major depressive disorder, bipolar disorder and schizophrenia. Mol Psychiatry. 27(4):1920–1935. 10.1038/s41380-022-01456-3 35194166 PMC9126816

[CIT0048] Meckel KR, Kiraly DD. 2019. A potential role for the gut microbiome in substance use disorders. Psychopharmacology (Berl). 236(5):1513–1530. 10.1007/s00213-019-05232-0 30982128 PMC6599482

[CIT0049] Medina-Rodriguez EM et al. 2020. Identification of a signaling mechanism by which the microbiome regulates Th17 cell-mediated depressive-like behaviors in mice. Am J Psychiatry. 177(10):974–990. 10.1176/appi.ajp.2020.19090960 32731813 PMC7647050

[CIT0050] Mendoza-Larios LA, García-Dolores F, Sánchez-Anguiano LF, Hernández-Tinoco J, Alvarado-Esquivel C. 2021. Association between suicide and *Toxoplasma gondii* seropositivity. Pathogens. 10(9):1094. 10.3390/pathogens10091094 34578127 PMC8466040

[CIT0051] National Center for Health. 2021. Provisional numbers and rates of suicide by month and demographic characteristics: United States. National Center for Health S, editor 10.15620/cdc:120830;2022.

[CIT0052] Nguyen TT et al. 2021. Gut microbiome in Schizophrenia: altered functional pathways related to immune modulation and atherosclerotic risk. Brain Behav Immun. 91:245–256. 10.1016/j.bbi.2020.10.003 33098964 PMC8023565

[CIT0053] Nguyen TT, Hathaway H, Kosciolek T, Knight R, Jeste DV. 2021. Gut microbiome in serious mental illnesses: a systematic review and critical evaluation. Schizophr Res. 234:24–40. 10.1016/j.schres.2019.08.026 31495702 PMC7056547

[CIT0054] Nickel T, Sonntag A, Backmund M, Pollmächer T. 2005. Depression during therapy with interferon α-how long should an antidepressant treatment last? Pharmacopsychiatry. 38(2):102–104. 10.1055/s-2005-837813 15744637

[CIT0055] Ohlsson L et al. 2019. Leaky gut biomarkers in depression and suicidal behavior. Acta Psychiatr Scand. 139(2):185–193. 10.1111/acps.12978 30347427 PMC6587489

[CIT0056] Olgiati P, Serretti A. 2023. In search of clinical targets for suicide prevention in major depressive disorder. Int Clin Psychopharmacol. 38(3):184–186. 10.1097/YIC.0000000000000468 36947411

[CIT0057] Peirce JM, Alviña K. 2019. The role of inflammation and the gut microbiome in depression and anxiety. J Neurosci Res. 97(10):1223–1241. 10.1002/jnr.24476 31144383

[CIT0058] Piton G et al. 2015. Enterocyte damage: a piece in the puzzle of post–cardiac arrest syndrome. Shock. 44(5):438–444. 10.1097/SHK.0000000000000440 26196845

[CIT0059] Ribeiro JD et al. 2016. Self-injurious thoughts and behaviors as risk factors for future suicide ideation, attempts, and death: a meta-analysis of longitudinal studies. Psychol Med. 46(2):225–236. 10.1017/S0033291715001804 26370729 PMC4774896

[CIT0060] Rifflet H et al. 1998. Suicidal impulses in patients with chronic viral hepatitis C during or after therapy with interferon alpha. Gastroenterol Clin Biol. 22(3):353–357. 9762223

[CIT0061] Rogers GB et al. 2016. From gut dysbiosis to altered brain function and mental illness: mechanisms and pathways. Mol Psychiatry. 21(6):738–748. 10.1038/mp.2016.50 27090305 PMC4879184

[CIT0062] Sang-Kap H. 2019. *Lactobacillus mucosae* and *Bifidobacterium longum* synergistically alleviate immobilization stress-induced anxiety/depression in mice by suppressing gut dysbiosis. J Microbiol Biotechnol. 29(9):1369–1374. 31564078 10.4014/jmb.1907.07044

[CIT0063] Severance EG et al. 2012. Complement C1q formation of immune complexes with milk caseins and wheat glutens in schizophrenia. Neurobiol Dis. 48(3):447–453. 10.1016/j.nbd.2012.07.005 22801085 PMC3465075

[CIT0064] Severance EG, Prandovszky E, Castiglione J, Yolken RH. 2015. Gastroenterology issues in schizophrenia: why the gut matters. Curr Psychiatry Rep. 17(5):27. 10.1007/s11920-015-0574-0 25773227 PMC4437570

[CIT0065] Sher L, Oquendo MA. 2022. Suicide: an overview for clinicians. Medical Clinics. 10.1016/j.mcna.2022.03.008 36402494

[CIT0066] Shkoporov AN, Turkington CJ, Hill C. 2022. Mutualistic interplay between bacteriophages and bacteria in the human gut. Nat Rev Microbiol. 20(12):737–749. 10.1038/s41579-022-00755-4 35773472

[CIT0067] Stevens BR et al. 2018. Increased human intestinal barrier permeability plasma biomarkers zonulin and FABP2 correlated with plasma LPS and altered gut microbiome in anxiety or depression. Gut. 67(8):1555–1557. 10.1136/gutjnl-2017-314759 PMC5851874 28814485

[CIT0068] Stone DM, Jones CM, Mack KA. 2021. Changes in suicide rates –United States, 2018-2019. MMWR Morb Mortal Wkly Rep. 70(8):261–268. 10.15585/mmwr.mm7008a1 33630824 PMC8344989

[CIT0069] Swannell SV, Martin GE, Page A, Hasking P, St John NJ. 2014. Prevalence of nonsuicidal self-injury in nonclinical samples: systematic review, meta-analysis and meta-regression. Suicide Life Threat Behav. 44(3):273–303. 10.1111/sltb.12070 24422986

[CIT0070] Tan RJ et al. 2015. Extracellular superoxide dismutase protects against proteinuric kidney disease. J Am Soc Nephrol. 26(10):2447–2459. 10.1681/ASN.2014060613 25644107 PMC4587687

[CIT0071] Vemuri R, Gundamaraju R, Eri R. 2017. Role of lactic acid probiotic bacteria in IBD. Curr Pharm Des. 23(16):2352–2355. 10.2174/1381612823666170207100025 28176664

[CIT0072] Wang J, Dunn AJ. 1998. Mouse interleukin-6 stimulates the HPA axis and increases brain tryptophan and serotonin metabolism. Neurochem Int. 33(2):143–154. 10.1016/s0197-0186(98)00016-3 9761458

[CIT0073] Wong M-L et al. 2016. Inflammasome signaling affects anxiety- and depressive-like behavior and gut microbiome composition. Mol Psychiatry. 21(6):797–805. 10.1038/mp.2016.46 27090302 PMC4879188

[CIT0074] Xiong R-G et al. 2023. The role of gut microbiota in anxiety, depression, and other mental disorders as well as the protective effects of dietary components. Nutrients. 15(14):3258. 10.3390/nu15143258 37513676 PMC10384867

[CIT0075] Yang J et al. 2020. Landscapes of bacterial and metabolic signatures and their interaction in major depressive disorders. Sci Adv. 6(49):eaba8555. 10.1126/sciadv.aba8555 33268363 PMC7710361

[CIT0076] Zajkowska I et al. 2024. Investigating the impacts of diet, supplementation, microbiota, gut-brain axis on schizophrenia: a narrative review. Nutrients. 16(14):2228. 10.3390/nu16142228 39064675 PMC11279812

[CIT0077] Zerekidze A et al. 2024. Impact of *Toxoplasma gondii* and human microbiome on suicidal behavior: a systematic review. J Clin Med. 13(2):593. 10.3390/jcm13020593 38276099 PMC10816148

[CIT0078] Zheng P et al. 2019. The gut microbiome from patients with schizophrenia modulates the glutamate-glutamine-GABA cycle and schizophrenia-relevant behaviors in mice. Sci Adv. 5(2):eaau8317. 10.1126/sciadv.aau8317 30775438 PMC6365110

