# Supplementary material for: The gut-brain axis and inflammatory mediators in suicide and mental disorders with high suicide rates: a review of current evidence
Source: Dialogues Clin Neurosci. 2026 Apr 10;28(1):145–56. doi: 10.1080/19585969.2026.2636468 (PMC13072704; doi:10.1080/19585969.2026.2636468)
Supplement: Supplemental Material [file TDCN_A_2636468_SM8829.zip › eTable 1.docx]

Table S1. The characteristics of studies investigating the associations between the gastrointestinal microbiome and inflammation with self-harm and suicide.

| Study | Design | Focus | Participants | Key outcome measures | Findings | Specific bacterial species identified | Limitations |
| --- | --- | --- | --- | --- | --- | --- | --- |
| Cai *et al.* (2022)^16^ | Case-Control Study | Gut microbiota and NSSI | 45 adolescents: 15 with NSSI, 15 with depression (without NSSI), 15 healthy controls | NSSI: SITBI-R. Depression: CDI. | Significant differences in gut microbiota diversity. Reduced species uniformity index (Shannon, Simpson). Specific taxa associations need further study. | Significant differences in beta diversity (Bray-Curtis, Jaccard). Exact species not well-defined in this summary. | Small sample size. Conducted in a specific region of China (limiting generalizability). Mechanisms underlying associations need further exploration. |
| Maes *et al.* (2023)^17^ | Cross-Sectional | Gut microbiome and suicidal behavior in depressed patients with ACEs | 142 patients: major depression and adverse childhood experiences | Suicidal behavior: MINI | Specific gut microbiomes inversely associated with suicidal behaviors: Clostridium, Butyricicoccus, Desulfovibrio piger, Parabacteroides merdae. Fusicatenibacter, Hungatellapositively associated. Enterotypes may contribute. | Clostridium cluster IV, Clostridium XIVa, Butyricicoccus, Desulfovibrio piger, Parabacteroides merdae, Fusicatenibacter, Hungatella | Cross-sectional (cannot infer causality). Homogenous sample (major depression and ACEs, limiting generalizability). Controlled for medication, but other lifestyle factors may influence. |
| Ahrens *et al.* (2022)^18^ | Cross-Sectional | Saliva microbiome and suicidal ideation | 181 university students | Suicidal ideation: BSSI | Saliva microbiome dominated by Veillonellain suicidal ideation. Megasphaerasignificantly higher. | Veillonella, Megasphaera | Cross-sectional (cannot infer causality). Relies on self-reported data (potential bias). University student population (limiting generalizability). |
| Mendoza-Larios *et al.* (2021)^19^ | Case-Control Study | Toxoplasma gondii and suicide | 89 suicide cases, 58 non-suicide controls | Suicide: official records. | No significant association between Toxoplasma gondii seropositivity (IgG) and suicide. | N/A | Low statistical power (may not detect a true association). Case-control (cannot infer causality). |
| Ohlsson *et al.* (2019)^25^ | Case-Control Study | Leaky gut biomarkers and depression/suicidal behavior | 30 patients: recent suicide attempts, 30 MDD, 30 healthy controls | Suicide attempts: intentional self-harm leading to hospitalization. Depression: MADRS. | Higher I-FABP, lower zonulin in recent suicide attempts vs. MDD and controls. IL-6 correlated positively with I-FABP, negatively with zonulin. | N/A | Small sample size (limiting power). Cross-sectional (prevents determining causality). Other factors (medication, lifestyle) could influence. |
| Almulla *et al.* (2023)^26^ | Case-Control Study | Immune profiles and first-episode depression/suicidal behavior | Subjects: first episode major dysmood disorder with suicidal behavior, healthy controls | Suicidal behavior: clinical assessment during first episode. | Suicidal behavior positively associated with platelet-derived growth factor, IL-16, M1 macrophages. Negatively associated with soluble IL receptor antagonists. | N/A | Preprint (interpret with caution). Small sample size. Focus on first-episode patients (limiting generalizability). |
| Lindqvist *et al.* (2009)^33^ | Case-Control Study | IL-6 levels in CSF and suicide attempts | 44 suicide attempters, 22 healthy controls | Suicide attempts: intentional self-harm leading to hospitalization. | IL-6 levels in CSF significantly higher in suicide attempters than controls. Violent attempters had highest IL-6. | N/A | Small sample size (particularly control group). Does not address confounding factors (medication, other conditions). |
| Lengvenyte *et al.* (2022)^34^ | Retrospective Study | Laxative misuse and suicidal behaviors in eating disorders | Patients with eating disorders | Suicide attempts: patient history. Suicidal ideation: BSSI. | Lifetime laxative misuse more likely to attempt suicide. Suicidal attempts in last 28 days reported more days of laxative misuse. Laxative use days in the past 28 days also associated with suicidal ideation. | N/A | Retrospective (relies on recall, potential bias). Eating disorder population (limiting generalizability). Cannot infer causality. |

Abbreviations: ACEs: Adverse Childhood Experiences; BSSI: Beck Scale for Suicidal Ideation; CDI: Children’s Depression Inventory; CNS: Central Nervous System; CSF: Cerebrospinal Fluid; I-FABP: Intestinal Fatty Acid Binding Protein; IFN: Interferon; IgG: Immunoglobulin G; IgM: Immunoglobulin M; IL-6: Interleukin-6; IL-16: Interleukin-16; MADRS: Montgomery-Åsberg Depression Rating Scale; MDD: Major Depressive Disorder; MINI: Mini-International Neuropsychiatric Interview; N/A: Not Applicable; NSSI: Non-Suicidal Self-Injury; OR: Odds Ratio; SITBI-R: Self-Injurious Thoughts and Behaviors Interview-Revised.
