# Supplementary material for: The gut-brain axis and inflammatory mediators in suicide and mental disorders with high suicide rates: a review of current evidence
Source: Dialogues Clin Neurosci. 2026 Apr 10;28(1):145–56. doi: 10.1080/19585969.2026.2636468 (PMC13072704; doi:10.1080/19585969.2026.2636468)
Supplement: Supplemental Material [file TDCN_A_2636468_SM8829.zip › eTable 2.docx]

Table S2. Key microbiome and inflammatory changes in mental disorders with high suicide rates.

| Mental disorder | Key microbiome changes | Inflammatory markers/mechanisms | Microbial metabolites | Mechanisms of microbiome influence | Clinical implications | Limitations | Future directions |
| --- | --- | --- | --- | --- | --- | --- | --- |
| MDD^37-40,43-48,51-56^ | ↑Proteobacteria, Bacteroidetes, Actinobacteria; ↓Firmicutes | ↑ TNF-α, IFN-γ, IL-6, IL-1; ↓ SCFAs (due to lower Firmicutes), leading to intestinal barrier dysfunction | LysoPC(16:0), LysoPC(20:0), deoxycholic acid, taurocholic acid, docosahexaenoic acid | Dysbiosis alters BBB permeability; vagus nerve activation; kynurenine pathway shifts serotonin synthesis | Probiotic strains (Bifidobacterium longum NK46, Lactobacillus mucosae NK41) inhibit TNF-α and NF-κB. Anti-inflammatory therapies targeting cytokines may reduce depressive symptoms. | Small sample sizes in studies; lack of diversity in participant populations; unclear causality between microbiota changes and MDD symptoms. | Investigate specific SCFA supplementation as a therapeutic approach; explore gut-brain axis interventions; assess long-term effects of probiotics on MDD outcomes;  conduct longitudinal studies to clarify causal relationships between dysbiosis and MDD development. |
|  | ↑Enterobacteriaceae, Eubacterium, Bilophila, etc.; ↓Faecalibacterium, etc. | TLR-4 activation by LPS from Gram-negative bacteria promotes depressive-like behaviors | SCFAs (butyrate) | Microglial activation; astrocyte atrophy; inflammatory cytokines increase neuroinflammation | Anti-inflammatory effects of SCFAs may improve gut barrier function and reduce systemic inflammation. | Limited understanding of specific bacterial species' roles in MDD pathogenesis. |  |
| Psychosis and schizophrenia^57-70^ | Alterations in Actinobacteria, Proteobacteria, Bacteroidetes, and Firmicutes | Altered metabolic pathways (e.g., Kdo2-lipid A biosynthesis); imbalance in oxidation/antioxidant system | Butyrate | Dysbiosis impacts CNS functions via inflammation and vagal nerve pathways | Probiotic supplementation (Lactobacilli, Bifidobacterium bifidum) improves PANSS scores. Complement C1q levels may serve as therapeutic targets for synaptic pruning-related symptoms. | Lack of longitudinal data; unclear causality between microbiome changes and symptoms; limited focus on non-bacterial microbiota like viruses or fungi. | Explore role of butyrate-producing bacteria in symptom management; investigate complement C1q inhibitors; study interactions between microbiota and antipsychotic medications. |
|  | ↑Prevotella, etc.; ↓Bacteroides, etc. | High levels of immune complexes with C1q influence synaptic pruning | Homocysteine | Immune complexes with C1q impact synaptogenesis; Helicobacter pylori infection influences dopaminergic dysfunction | Reducing homocysteine levels may improve cognitive function and reduce inflammation. Addressing latent Toxoplasma gondii infections may mitigate negative symptoms associated with schizophrenia. | Limited research on specific probiotic strains for schizophrenia treatment. | Investigate individualized microbiome-based therapies for schizophrenia patients based on their unique microbial profiles. |
| Anxiety disorders^50,71,72,85^ | ↑Paraprevotella, Caldivirga, etc.; ↓Faecalibacterium, Eubacterium rectale | Endotoxemia leads to neuroinflammation through cytokine migration across the BBB or afferent nerve signaling | 4-EPS | Stress-induced dysbiosis triggers endotoxemia and neuroinflammation | Administration of Bacteroides fragilis reduces neurotoxic metabolites like 4-EPS and improves gut permeability and anxiety-like behavior. | Few studies focus on anxiety-specific microbiome changes compared to other disorders like MDD or schizophrenia. | Expand research on targeted interventions (e.g., specific probiotics) for anxiety disorders; study the role of gut permeability markers in anxiety pathogenesis. |
| SUD^56,58,62,73-77^ | ↓ Firmicutes (Lactobacilli,Enterococci), Actinobacteria (Bifidobacteria) in alcohol users; ↑ Bacteroidetes | Gut permeability alterations lead to systemic inflammation | LPS | Alcohol increases gut permeability via LPS production; dysbiosis alters microglial activity | Anti-inflammatory therapies targeting LPS-mediated pathways may reduce systemic inflammation and improve behavioral responses to drugs. | Lack of diversity in SUD populations studied (e.g., alcohol vs methamphetamine users); limited understanding of how dysbiosis varies by substance type. | Study microbiome differences across various substances (e.g., alcohol vs opioids); develop interventions targeting gut permeability restoration in SUD patients. |

Abbreviations: 4-EPS: 4-Ethylphenylsulfate; BBB: Blood-Brain Barrier; CNS: Central Nervous System; GAD: Generalized Anxiety Disorder; HPA: Hypothalamic-Pituitary-Adrenal; IDO: Indoleamine-2,3-Dioxygenase; IFN-γ: Interferon-gamma; IL: Interleukin; LPS: Lipopolysaccharides; MDD: Major Depressive Disorder; MUD: Methamphetamine Use Disorder; NF-κB: Nuclear Factor Kappa B; PANSS: Positive and Negative Syndrome Scale; SCFA: Short-Chain Fatty Acid; SUD: Substance Use Disorder; TLR-4: Toll-Like Receptor 4; TNF-α: Tumor Necrosis Factor-alpha; H. pylori: Helicobacter pylori; T. gondii: Toxoplasma gondii.
